# Mitochondrial encephalomyopathy, lactic acidosis, and stroke-like episodes syndrome: a case report from Nepal

**DOI:** 10.1097/MS9.0000000000000712

**Published:** 2023-05-03

**Authors:** Ram C. Subedi, Raju Paudel, Sharma Paudel, Lekhjung Thapa, Subash Phuyal, Naresh kharbuja, Ayush Adhikari

**Affiliations:** a Department of Neurology, Grande International Hospital; b Department of Neurology, National Institute of Neurological and Allied Sciences; c Department of Neurology, Shree Birendra Hospital

**Keywords:** case report, lactic acidosis, MELAS, MIDD, mitochondriopathies

## Abstract

Mitochondrial encephalomyopathy, lactic acidosis, and stroke-like syndrome (MELAS) is a rare neurodegenerative inherited disorder that is characterized by stroke-like episodes, seizures, endocrine, and multiple system involvement. It is important to consider it as a differential diagnosis in a young patient with stroke-like episodes as it is progressive and has multiple complications.

**Case presentation:**

A 28-year-old male presented with slurring of speech and drowsiness for 7 h. He was a diagnosed case of type 2 diabetes mellitus, Wolf-Parkinson-White syndrome, and bilateral hearing loss.

**Clinical findings and investigations:**

The patient had expressive aphasia with impaired fluency, repetition, and naming. After being discharged, he represented with loss of consciousness and involuntary movements of the whole body. MRI and MRS showed extension of hyperintense lesions to parieto-occipital regions from temporal regions not limited by vascular territories. MELAS was considered, which was confirmed by molecular genetic analysis. Coenzyme Q10 was used for MELAS. Insulin, Linagliptin, and levetiracetam were used for diabetes and seizures. Regular follow-up was advised to the patient.

MELAS is an important syndrome to consider in any young patient presenting with unexplained stroke disorders. A high index of suspicion is needed in an appropriate clinical setting to avoid misdiagnosis.

## Introduction

Highlights

- Mitochondrial encephalomyopathy, lactic acidosis, and stroke-like syndrome is a rare neurodegenerative, inherited mitochondrial disorder that involves multiple organs.
- Symptoms and signs typically comprise mitochondrial myopathy, encephalopathy with stroke-like episodes, seizures and/or dementia, and lactic acidosis.
- Other common symptoms include cardiomyopathy, progressive (bilateral) sensorineural hearing loss, migraine-like headache, recurrent vomiting, peripheral neuropathy, ophthalmoplegia, pigmentary retinopathy, diabetes, hypoparathyroidism, ataxia, and short stature.
- Ragged-red fibers are the hallmark feature of muscle biopsy. Molecular genetic analysis can show the underlying genetic defects.
- There is no curative treatment for mitochondrial encephalomyopathy, lactic acidosis, and stroke-like syndrome. L-arginine and coenzyme Q10 are used while complications are managed accordingly with a multidisciplinary team.

Mitochondriopathies are multisystem disorders with significant morbidity and is associated with broad phenotypic diversity. The protean manifestations include asymptomatic carrier state, cardiac conduction defects, hearing impairment with or without diabetes, maternally inherited diabetes and deafness and mitochondrial encephalomyopathy, lactic acidosis, and stroke-like syndrome (MELAS)^1^. MELAS is the most common subtype of mitochondrial encephalopathy and is still a very rare genetic disorder. MELAS is most commonly caused by point mutations at the A3243G of the tRNA LEU gene in the mitochondrial DNA, transmitted by maternal mode of inheritance^2^. MELAS usually presents in childhood with short stature, seizure disorder, stroke-like events, diabetes mellitus, hearing loss, and lactic acidosis. Vascular infarcts are uncommon in young patients, hence, awareness of MELAS presenting as an infarct in young patients could lead to a more timely and accurate diagnosis of this condition as in our case.

We reported this case following the updated consensus-based Surgical Case Report (SCARE) Guidelines^3^.

## Case report

A 28-year-old man presented to the emergency department with complaints of sudden onset of slurring of speech and drowsiness for 7 h. The patient had prior diagnoses of type 2 diabetes mellitus, Wolf-Parkinson-White syndrome, and bilateral hearing loss for 3 years. His mother and elder sister also had Type 2 Diabetes mellitus and bilateral hearing loss. For hearing loss, both the patient and his sister were using hearing aids. The general body examination was normal without abnormalities. Vitals were stable, with a pulse of 103 bpm, blood pressure of 110/80 mmHg, respiratory rate of 22 bpm, and oxygen saturation of 99% in room air. Systematic examination of the patient showed normal respiratory, cardiovascular, and abdominal findings. The patient had a Glasgow Coma Scale score of 15. There were no signs of meningeal irritation and other focal neurological deficits. He could understand the gesture and follow the commands but had expressive aphasia with impaired fluency, naming, and repetition.

**Figure 1:**
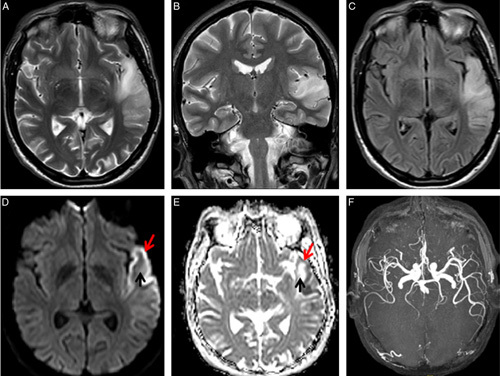
MRI Brain showing T2/FLAIR hyperintensity involving the left superior temporal region and insular cortex showing restriction on diffusion weighted imaging. MR angiogram showing normal flow related enhancement without any occlusion.

The laboratory investigations of the patient showed a cell count of 11760 /mm^3^ with platelets 228000 /mm^3^. Electrolytes were normal with sodium 137 and potassium 4.4. The hemoglobin level was measured as 14 gm/dl. Cardiac enzymes were normal. Arterial blood gas analysis showed lactate of 2.6 mmol/l , pH of 7.32, pCO_2_ 33.2 mmHg, pO_2_ 99.4 mmHg, bicarbonate level of 18.1 mmol/l. The CSF lactate level was 7.3 mmol/l. The electrocardiogram showed normal sinus rhythm, with no delta waves. The echocardiogram was normal with an ejection fraction of 62%. The carotid Doppler ultrasound study was normal. An audiogram revealed bilateral moderate sensorineural hearing loss. MRI with angiography of the head revealed T2/FLAIR hyperintensity involving the left superior temporal region and insular cortex showing restriction on diffusion weighted imaging (Fig. 1). MR angiography showed normal flow related enhancement of intracranial vessels. Based on the findings, he was diagnosed as maternally inherited diabetes and deafness, Wolf-Parkinson-White syndrome, and stroke. The patient was admitted and managed with insulin, aspirin, statin, and supportive therapy. The patient was subsequently discharged after improvement.

**Figure 2:**
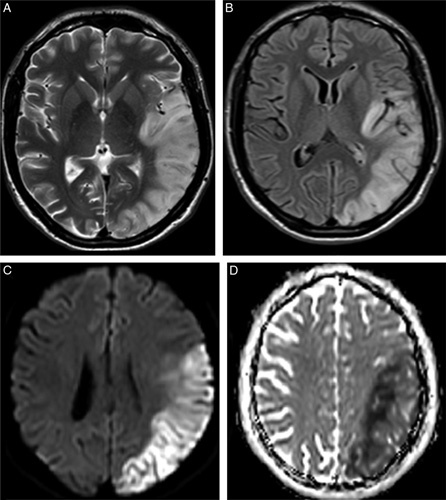
MRI Brain showing T2/FLAIR hyperintensities with extension of infarct in the occipital region with diffusion restriction.

A day after discharge, the patient again presented to the emergency department with complaints of sudden loss of consciousness with abnormal, involuntary jerky movements of all limbs along with uprolling of the eyes, frothing, and a tongue bite an hour back as described by his relative. On neurological examination, the patient also had weakness of the right upper limb and difficulty in comprehension in addition to expressive aphasia from the prior stroke. On examination, there was global aphasia with 4/5 power of right upper limb. A repeat MRI and Magnetic resonance spectroscopy (MRS) was performed, which showed an extension of the infarct in the occipital region (nonterritorial) (Fig. 2). MRS showed a lactate doublet peak with a relative decrease in N-acetylaspartate when sampling was performed over the areas of cortical abnormality. MELAS was deemed a likely explanation for his multi-systemic disease and further genetic testing was sent. Due to the unavailability of the genetic analysis techniques in the country, the genetic analysis was done abroad, which revealed the presence of the MELAS A3243G mitochondrial mutation in a heteroplasmic state, which confirmed the diagnosis of MELAS. Molecular genetic analysis revealed the presence of the MELAS A3243G mitochondrial mutation in a heteroplasmic state, which confirmed the diagnosis of MELAS. The patient was then discharged after two weeks on insulin, linagliptin, levetiracetam, and coenzyme Q.

## Discussion

**Figure 3:**
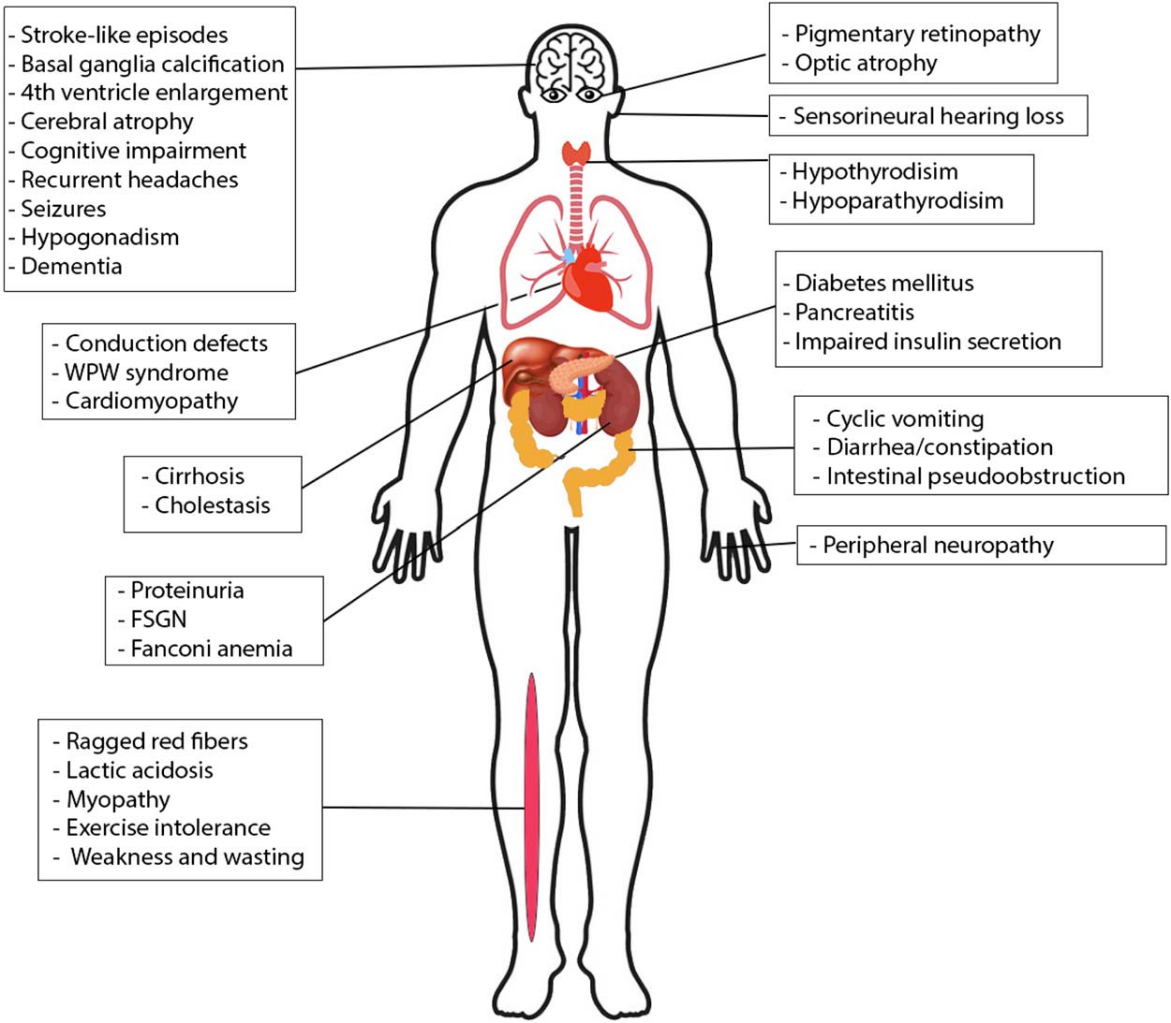
Multi-system involvement in MELAS.

MELAS is a rare neurodegenerative, inherited mitochondrial disorder that involves multiple organs. It usually presents in the first or second decade of life though an adult onset form is now recognized^4^. Symptoms and signs typically comprise mitochondrial myopathy, encephalopathy with stroke-like episodes, seizures and/or dementia, and lactic acidosis^5^. Other symptoms include cardiomyopathy, progressive (bilateral) sensorineural hearing loss, migraine-like headache, recurrent vomiting, peripheral neuropathy, ophthalmoplegia, pigmentary retinopathy, diabetes, hypoparathyroidism, ataxia, and short stature^6,7^ (Fig. 3). Despite the clinical heterogeneity of this disorder, nonischemic stroke-like episodes are considered defining features^2,8^.

On cerebral imaging, characteristic radiological findings include bilateral basal ganglia calcification, focal hypodense lesions, enlargement of the fourth ventricle, and generalized atrophy^9,10^. MRI findings of cortical and subcortical lesions that cross vascular territories in a patient with stroke-like symptoms should raise suspicion for a mitochondrial disorder and consideration of MRS. MRS provides an important diagnostic adjunct, along with serum and CSF lactate during acute episodes and respiratory enzyme defects in skeletal muscle biopsy samples^11^. However, the above-mentioned radiological features are not specific for MELAS and clinical correlation is always required. The notable diseases whose imaging may mimic imaging findings in MELAS include herpes encephalitis, posterior reversible encephalopathy syndrome, progressive multifocal leukoencephalopathy, and subacute ischemic strokes^12–15^. ‘Ragged-red fibers’ are the hallmark feature in the muscle of most patients. However, mutation load in urinary epithelial cells has shown to correlate well with disease activity, obviating the need for muscle biopsy in many cases^16^.

As with other mitochondrial diseases, there is no curative treatment for MELAS. Treatment options are limited and supportive therapy along with genetic counseling are important component of patient management. Common medication that are currently used include antioxidants, respiratory chain substrates, and cofactors in the form of vitamins. Coenzyme Q_10_ is commonly prescribed^17^. L-arginine has been shown to be effective at treatment and prevention of stroke-like episodes^18^. Considerations are required for drugs that can cause lactic acidosis and those that can act as mitochondrial toxins. Metformin can exacerbate lactic acidosis and so alternative medications should be used for the treatment of diabetes^19^. Valproate and other drugs like aminoglycosides, linezolid with potential mitochondrial toxicity should also be avoided^20^.

The disease progresses over years with episodic deterioration related to stroke-like events while dysfunction in daily living, motor activity, and cognition occur more rapidly. Patients with disease onset before 18 years of age are reported to have a higher mortality rate^8^. Regular assessment for other organ involvement, management of complications, and family screening is recommended. Knowledge of this condition and its variable presentation should guide appropriate radiological and biochemical investigations, genetic analysis, and if needed invasive muscle biopsy to avoid misdiagnosis.

## Conclusion

MELAS is a rare, inherited, and progressive disease with multiple organ system involvement. Awareness of the disease as a differential is required for any stroke-like episodes in a young patient. Timely diagnosis, genetic counseling, whole body assessment, and regular follow-up is important to improve the quality of life of affected patients.

## Ethical approval

NA.

## Consent

Written informed consent was obtained from the patient for publication of this case report and accompanying images. A copy of the written consent is available for review by the Editor-in-Chief of this journal on request.

## Source of funding

None.

## Author contribution

R.C.S., R.P.: led data collection, contributed in writing the case information, and discussion; S.P., L.T.: contributed to the process of original draft preparation and introduction; S.P., N.K.: conceptualization, methodology, and discussion; A.A.: revised it, contributed in review, and editing, edited the rough draft into the final manuscript.

## Conflicts of interest disclosure

No conflict of interest.

## Research registration unique identifying number (UIN)

Not applicable.

## Guarantor

Correspondence: Dr. Ram Chandra Subedi, Grande International Hospital, 44600 Kathmandu, Nepal. Tel: +977 985 127 9838. E-mail: manohar.rcs@gmail.com.

## Provenance and peer review

Not commissioned, externally peer reviewed.

## Data availability statement

Not applicable.
